# A Survey on Oral Health Knowledge, Attitudes and Practices of Pregnant Women Attending Four General Health Hospitals in Switzerland

**DOI:** 10.3290/j.ohpd.b2573007

**Published:** 2022-01-20

**Authors:** Ioanna Lazaridi, Alkisti Zekeridou, Leandra Schaub, Daniela Prudente, Maria Razban, Catherine Giannopoulou

**Affiliations:** a Dentist, Research and Teaching Assistant, Division of Regenerative Dentistry and Periodontology, University Clinics of Dental Medicine, University of Geneva, Geneva, Switzerland. Wrote the manuscript, performed the survey, data and statistical analysis, protocol and study design. *These authors contributed equally to this article.; b Periodontist, Research and Teaching Fellow, Division of Regenerative Dentistry and Periodontology, University Clinics of Dental Medicine, University of Geneva, Geneva, Switzerland. Wrote the manuscript, performed the survey, data and statistical analysis, protocol and study design. *These authors contributed equally to this article.; c Dentist, Research and Teaching Assistant, Division of Orthodontics, University Clinics of Dental Medicine, University of Geneva, Geneva, Switzerland. Performed the survey, data accumulation and analysis, protocol design.; d Dentist, Research and Teaching Assistant, Division of Cariology and Endodontology, University Clinics of Dental Medicine, University of Geneva, Geneva, Switzerland. Performed the survey, data accumulation and analysis, protocol design.; e Dentist, Private Practice, Bulle, Switzerland. Performed the survey, data accumulation and analysis, protocol design.; f Professor, Division of Regenerative Dentistry and Periodontology, University Clinics of Dental Medicine, University of Geneva, Geneva, Switzerland. Wrote the manuscript, performed the survey and statistical analysis, protocol and study design, supervised and coordinated the project

**Keywords:** education, oral health, pregnancy, self-care

## Abstract

**Purpose:**

To evaluate the knowledge and practices of Swiss women regarding oral health during pregnancy.

**Materials and Methods:**

Self-reported questionnaires were attributed to 385 women from 4 public hospitals in the French speaking part of Switzerland from February 2015 to June 2016.The questionnaire consisted of 32 questions including demographic characteristics, oral health habits and awareness of oral changes during pregnancy.

**Results:**

The majority of women (64%) were in the 3rd trimester of pregnancy and had a university education (41%). Oral health was considered very important for half of the women (52%) and moderately important for 38% of them. 71% of the women did not notice any change concerning their oral health conditions. Of the remaining 29%, gingival bleeding was the main symptom reported, followed by gingival redness and oedema. Pain and sensitivity were also reported by a few participants. Most of the pregnant women attended dental appointments during their pregnancy, but not on a regular basis, mainly because of lack of time. Almost half of the population had an adequate oral hygiene routine and adapted their eating habits to a much healthier pattern during pregnancy. A large proportion of the participants (71%) was aware that pregnancy renders teeth and gums more vulnerable and that oral health is related to adverse pregnancy outcomes. However, this information was rarely imparted to them by health professionals.

**Conclusion:**

Although pregnant women in the French speaking part of Switzerland seem to be moderately informed about the importance of oral health during pregnancy, health-care professionals do not seem to participate actively. Health professionals need to more actively inform pregnant patients about the importance of preventive oral health measures and oral health care during pregnancy.

Pregnancy is a period in which important physical, biological and hormonal transformations occur in the body of the woman. During the 38–42 weeks of gestation, systemic changes affect multiple systems and organs, such as the endocrine and respiratory systems as well as the oral cavity.^18^ In turn, pregnancy can be affected by several conditions and pathologies of the mother, such as obesity, high blood pressure, and chronic diseases, e.g. periodontal disease.^6,23,31,37^ Comorbidities and inflammatory status increase the risk of complications, for instance, preterm infants, low birth weight, spontaneous abortion and pre-eclampsia.^28^ The effect of the hormonal changes on oral health occurring during pregnancy has attracted much attention. During pregnancy, the estrogen level increases, resulting in higher prevalence of oedema, exacerbation of gingival bleeding and increased gingival sensitvity. Pregnancy can aggravate a pre-existent inflammatory state of the gums. The severity of untreated gingivitis and periodontitis may increase significantly prenatally.^10^ Gingivitis can affect 36%–100% of pregnant women, and although bacterial plaque is always the cause, hormonal changes act as modifying factors.^21^

In recent decades, great efforts have been made to better understand the effect of the gingival status on pregnancy complications. The plausible mechanisms linking these two conditions are the systemic dissemination of periodontal bacteria and the entrance of the locally-produced pro-inflammatory cytokines to the systemic circulation.^17^ In fact, current evidence shows that nonspecific inflammatory mediators, such as tumor necrosis factor alpha (TNF-α), interleukin-1 beta (IL-1β) and prostaglandin E2 (which plays an important role in the initiation of birth), can also be prematurely induced in periodontitis. Furthermore, periodontal pathogens and their virulence factors can have a direct effect on the fetus/placenta unit, where they can prematurely increase the levels of inflammatory cytokines. Thus, both local and systemic inflammation, as well as augmentation of C-reactive protein (CRP), occur in the plasma, resulting in premature rupture of the placenta.^30^ A study by Gupta et al^15^ identified greater amounts of periodontal pathogens – for instance *Porphyromonas gingivalis* (Pg), *Tannerella forsythia* (Tf), *Aggregatibacter actinomycetemcomitans* (Aa) and *Treponema denticola* (Td) – in mothers of preterm infants compared to mothers of normal-weight infants.

The importance of oral health maintenance, education and treatment during pregnancy^20,25,27^ is now evident. Many studies have been conducted in the form of surveys concerning knowledge, attitudes and dental care practices during pregnancy. These surveys were addressed to dental practitioners,^8,13,14,19^ dental hygienists,^5,34^ gynecologists and obstetricians,^7^ and pregnant women as well. In all studies, the need for better education and knowledge on dental care and related pregnancy risks was emphasised.^1,15,22,24,29^ Recently, our group conducted a similar survey among Swiss dentists to evaluate their practices concerning oral care during pregnancy. The results showed that there is good level of knowledge related to potential oral health risks during pregnancy; however, a need for clearer guidelines and directions on treatment protocols was expressed by the majority of the participants.^33^ In the present survey, our hypothesis was that many women are not sufficiently aware of the bi-directional relationship between oral health and pregnancy. Thus, we aimed to evaluate the knowledge and attitudes regarding the oral health of pregnant women who were followed-up at four Swiss public hospitals.

## Materials and Methods

### Sample and Data Collection

A total of 385 pregnant women agreed to participate in the present cross-sectional survey. The sample size consisted of women who attended four general health hospitals located in the French-speaking part of Switzerland: University Hospital of Geneva (HUG), University Hospital of Lausanne (CHUV), Hospital of Riviera-Chablais Vaud-Valais (HRC) and Cantonal Hospital of Fribourg (HFR). Data were collected at 2 time points. The first cohort included subjects attending the HUG and the HPC, while the second cohort comprised those attending the CHUV and HFR. The questionnaires were distributed in person either by the secretary or by the head nurse-midwife upon arrival in the waiting area. Once the questionnaires were filled out, they were placed in a ballot box, which the authors collected at regular intervals. Written informed consent was obtained from the subjects. Recruitment was undertaken until there was a sufficient number of participants. The inclusion criteria for participation were: age >18 years with confirmed pregnancy status and understanding of the French language. The investigation was carried out according to the rules of the Declaration of Helsinki of 1975, revised in 2013.

### Questionnaire

The questionnaire consisted of 32 questions, the majority of which were adapted from similar previous studies.^12,24,30^ The survey questions were prepared by a panel of dental professionals and were validated by the University-Hospital Clinical Research Center of the University of Geneva. The questionnaire included general information on the age, highest completed educational level, whether it is the first pregnancy, and the pregnancy trimester. The participants were also asked to describe their oral health habits, whether they modified certain attitudes during pregnancy (smoking, alcohol) and whether they noticed any changes in their oral cavity since the beginning of their pregnancy. They completed questions on whether they were provided with sufficient information on the effect of pregnancy on oral health and on possible complications related to the birth. Finally, women were asked to rate the importance of oral health on a ten-point scale from ‘very important’ to ‘not important’.

### Statistical Analysis

We used descriptive statistics with frequency and percentages to analyse the various questions answered by the pregnant women.

## Results

The sample size was 385 women with an age range between 19 and 43 years attending the four Swiss hospitals named above. The first cohort (n = 198) included 98 women attending the HUG and 100 attending the HFR for consultation. The second cohort (n = 187) included 155 attending the CHUV and 32 attending the HRC for consultation.

The demographic and pregnancy characteristics are presented in Table 1. Approximately 73% of the population were between 22 and 35 years old and 16.6% were over 35. Only 17 women were younger than 22 years at the time the questionnaire was given. While 55% of the participants had no children at the time of the survey (excluding fetus), 45% had one or more children. 41% were university graduates, followed by 27% who were highschool graduates. The majority of women (64%) were in the 3rd trimester of pregnancy.

**Table 1:** Demographics

Clinical parameter	Total (n = 385)	Cohort 1 (n = 198)	Cohort 2 (n = 187)
**Age**	**19–43 years old**	**19–42 years old**	**21–43 years old**
Up to 21 years old	17 (4.4)	9 (4.5)	8 (4.3)
22–35 years old	282 (73.3)	138 (69.7)	144 (77.0)
More than 35 years	64 (16.6)	32 (16.2)	32 (17.1)
No answer	22 (5.7)	19 (9.6)	3 (1.6)
**Educational level**			
University	159 (41.3)	74 (37.4)	85 (45.4)
Secondary school	103 (26.7)	68 (34.3)	35 (18.7)
Primary school	18 (4.7)	13 (6.6)	5 (2.7)
Apprenticeship CFC*	54 (14.0)	6 (3.0)	48 (25.7)
Maturité fédérale**	13 (3.4)	2 (1.0)	11 (5.9)
No answer	38 (9.9)	35 (17.7)	3 (1.6)
**1st pregnancy**			
Yes	211 (54.8)	115 (58.1)	96 (51.3)
No	173 (44.9)	83 (41.9)	90 (48.2)
No answer	1 (0.3)	0 (0)	1 (0.5)
**Number of children**			
0	214 (55.6)	115 (58.1)	99 (53.0)
1	100 (26.0)	41 (20.7)	59 (31.5)
2	55 (14.3)	33 (16.7)	22 (11.8)
3	15 (3.9)	9 (4.5)	6 (3.2)
No answer	1 (0.2)	0 (0)	1 (0.5)
**Trimester of pregnancy**			
1st	36 (9.4)	23 (11.6)	13 (7.0)
2nd	99 (25.7)	54 (27.3)	45 (24.1)
3rd	246 (63.9)	121 (61.1)	125 (66.8)
No answer	4 (1.0)	0 (0)	4 (2.1)
**Smokers**	53 (13.8)	29 (14.6)	24 (12.8)

Total number of patients: 385; cohort 1: hospitals of Fribourg and Geneva, number of patients: 198; cohort 2: hospitals of Lausanne and Valais, number of patients: 187. *Apprenticeship for federal certificate of capacity (CFC); **Swiss federal highschool diploma.

Oral health was considered very important (score 9-10 on the VAS scale) by half of the women (52%) and moderately important by 38% (Table 2). In an additional question addressed only to the second cohort, it was reported that the perceived importance of oral health did not change during pregnancy. Almost 71% of the women did not notice any change in their oral health condition. Of the remaining 29%, bleeding was the main symptom reported, followed by gingival redness and oedema. Pain and sensitivity were reported by almost 11% and 12% of participants, respectively.

**Table 2:** Changes in oral health

Questions	Total (n = 385)	Cohort 1 (n = 198)	Cohort 2 (n = 187)
**During your pregnancy have you noticed any changes in your oral health?**			
**Yes**	110 (28.6)	42 (21.2)	68 (36.3)
Bleeding	81 (73.6)	26 (61.9)	55 (80.9)
Oedema, gingival redness	25 (22.7)	0 (0)	25 (36.8)
Pain, toothache	12 (10.9)	3 (7.1)	9 (13.2)
Tooth migration	1 (0.9)	0 (0)	1 (1.5)
Sensitivity, gingival sensitivity	13 (11.8)	12 (28.6)	1 (1.5)
Caries	0 (0)	0 (0)	0 (0)
Infection, abcess	2 (1.8)	1 (2.4)	1 (1.5)
No answer	4 (3.7)	0 (0)	4 (5.9)
**No**	273 (70.9)	156 (78.8)	117 (62.6)
No answer	2 (0.5)	0 (0)	2 (1.1)
**How important is oral health to you on a scale of 1 to 10?**			
0 to 5	30 (7.8)	6 (3.1)	24 (12.8)
6 to 8	148 (38.4)	66 (33.3)	82 (43.9)
9 to 10	201 (52.2)	126 (63.6)	75 (40.1)
No answer	6 (1.6)	0 (0)	6 (3.2)

Total number of patients: 385; cohort 1: hospitals of Fribourg and Geneva, number of patients: 198; cohort 2: hospitals of Lausanne and Valais, number of patients: 187.

As shown in Table 3, more than half of the participants visited their dentist for a routine check-up; however, only approximately one-third of them had consulted their dentist the last 6 months. During pregnancy, 83% of women did not consult their dentist. The reasons for not attending a dental check-up were the absence of dental symptoms (29%), lack of time due to too many appointments (8%), and lack of money (8%). However, a large proportion of women gave no reason for not consulting a dentist during pregnancy (37.4%). 58% of the participants reported brushing their teeth twice per day and almost 20% brushed 3 times per day. Less than half (45%) used interdental cleaning aids. These habits (frequency of brushing and interdental cleaning aids) did not change during pregnancy for the majority of the women (93%). Only 14% of the participants were smokers and 6% drank alcohol regularly (on a daily basis). During pregnancy, smoking decreased in half of the women. Finally, the eating habits changed in one-third of the participants (34%), towards consuming more fruits and vegetables, decreasing coffee and avoiding raw foods to avoid the risk of toxoplasmosis.

**Table 3:** Habits and dental check-ups during pregnancy

Questions	Total n (%)	Cohort 1 n (%)	Cohort 2 n (%)
**Are visits to the dentists part of your routine check-ups?**			
**Yes**	201 (52.2)	100 (50.5)	101 (54.0)
No	181 (47.0)	98 (49.5)	83 (44.4)
No answer	3 (0.8)	0 (0)	3 (1.6)
**When was your last check-up?**			
<3 months ago	63 (16.3)	26 (13.1)	37 (19.8)
>3 months to <6 months ago	50 (13.0)	21 (10.6)	29 (15.5)
>6 months to <1 year ago	78 (20.3)	35 (17.7)	43 (23.0)
>1 year ago	102 (26.5)	62 (31.3)	40 (21.4)
No answer	92 (23.9)	54 (27.3)	38 (20.3)
**Did you consult your dentist during pregnancy?**			
**Yes**	62 (16.1)	39 (19.7)	23 (12.3)
No (more than one answer is possible)	321 (83.4)	158 (79.8)	163 (87.2)
No problems	93 (29.0)	64 (40.5)	29 (17.8)
Frequent appointments	27 (8.4)	6 (3.8)	21 (12.9)
No money	27 (8.4)	19 (12.0)	8 (4.9)
It didn’t occur to me	6 (1.9)	0 (0)	6 (3.7)
Consulted a dentist abroad	5 (1.6)	1 (0.6)	4 (2.5)
Waited until after birth	7 (2.2)	3 (1.9)	4 (2.5)
Not important	23 (7.1)	17 (10.8)	6 (3.7)
No time	11 (3.4)	8 (5.1)	3 (1.8)
Didn’t want to	3 (0.9)	2 (1.3)	1 (0.6)
Had many other appointments	1 (0.3)	0 (0)	1 (0.6)
Don’t like the dentist	2 (0.6)	1 (0.6)	1 (0.6)
No answer	120 (37.4)	37 (23.4)	83 (50.9)
**No answer**	2 (0.5)	1 (0.5)	1 (0.5)
**How many times a day do you brush your teeth?**			
1	39 (10.1)	24 (12.1)	15 (8.0)
2	224 (58.2)	133 (67.2)	91 (48.6)
2 to 3	33 (8.6)	11 (5.6)	22 (11.8)
3	76 (19.8)	27 (13.6)	49 (26.2)
3 to 4	4 (1.0)	1 (0.5)	3 (1.6)
4	4 (1.0)	2 (1.0)	2 (1.1)
No answer	5 (1.3)	0 (0)	5(2.7)
**Has your brushing frequency increased since you became pregnancy?**			
Yes	24 (6.2)	3 (1.5)	21 (11.2)
No	358 (93.0)	195 (98.5)	163 (87.2)
No answer	3 (0.8)	0 (0)	3 (1.6)
**Do you use interdental hygiene products?**			
Yes	172 (44.7)	69 (34.8)	103 (55.1)
No	209 (54.3)	129 (65.2)	80 (42.8)
No answer	4 (1.0)	0 (0)	4 (2.1)
**Are you a smoker?**			
Yes	53 (13.8)	29 (14.6)	24 (12.8)
No	327 (84.9)	169 (85.4)	158 (84.5)
No answer	5 (1.3)	0 (0)	5 (2.7)
**Do you drink alcohol regularly?**			
Yes	22 (5.7)	12 (6.1)	10 (5.3)
No	358 (93.0)	186 (93.9)	172 (92.0)
No answer	5 (1.3)	0 (0)	5 (2.7)
**During your pregnancy, did you change your daily habits? (HB, food, tobacco, alcohol, other)**			
Yes	184 (47.8)	50 (25.2)	134 (71.6)
No	193 (50.1)	148 (74.8)	45 (24.1)
No answer	8 (2.1)	0 (0)	8 (4.3)
**Since becoming pregnant, have you smoked less?**			
Yes	26 (49.1)	12 (41.4)	14 (58.3)
No	24 (45.3)	17 (58.6)	7 (29.2)
No answer	3 (5.7)	0 (0)	3 (12.5)
**Since becoming pregnant, has your alcohol consumption decreased?**			
Yes	108 (28.1)	17 (8.6)	91 (48.7)
No	108 (28.1)	17 (8.6)	91 (48.7)
No answer	169 (43.8)	164 (82.8)	5 (2.6)
**Did you change your eating habits during pregnancy?**			
Yes	130 (33.8)	26 (13.1)	104 (55.6)
No	250 (64.9)	172 (86.9)	78 (41.7)
No answer	5 (1.3)	0 (0)	5 (2.7)

Total number of patients: 385; cohort 1: hospitals of Fribourg and Geneva, number of patients: 198; cohort 2: hospitals of Lausanne and Valais, number of patients: 187.

Table 4 shows the participants’ knowledge and awareness on the importance of oral diseases during pregnancy. 71% of the sample population were aware that pregnancy renders teeth and gums more vulnerable. However, this information was given by obstetricians, gynecologists, or midwives to only 29% of the participants. Brochures in the waiting room were the main source of knowledge for 59% of the participants. Finally, regarding the potential influence of oral health on adverse pregnancy outcomes, only half of the pregnant women were aware of the link between oral health and pregnancy complications. The sources of information reported were the doctor/dentist, internet, magazines and family/friends.

**Table 4:** Knowledge and information concerning oral health related risks during pregnancy

Questions	Total n (%)	Cohort 1 n (%)	Cohort 2 n (%)
**Has the dentist or gynecologist informed you about the importance of oral hygiene during pregnancy?**			
Yes	112 (29.1)	42 (21.2)	70 (37.4)
No	267 (69.3)	153 (77.3)	114 (61.0)
No answer	6 (1.6)	3 (1.5)	3 (1.6)
**Do you think you have received enough information about the importance of oral hygiene during pregnancy? (from the gynecologist, dentist, brochures, internet)**			
Yes		NR	67 (35.8)
No		NR	111 (59.4)
No answer		NR	9 (4.8)
**In the waiting area, do you read various brochures containing information and advice about pregnancy?**			
Yes	227 (59.0)	114 (57.6)	113 (60.4)
No	156 (40.5)	84 (42.4)	72 (38.5)
No answer	2 (0.5)	0 (0)	2 (1.1)
**If yes, have you read about the association between pregnancy and oral problems?**			
Yes		NR	45 (39.8)
No		NR	67 (59.3)
No answer		NR	1 (0.9)
**If yes, are you aware that poor oral hygiene can lead to complications during pregnancy?**			
Yes (more than one answer is possible)	192 (49.9)	102 (51.5)	90 (48.1)
Doctor/dentist	53 (27.6)	24 (23.5)	29 (32.2)
Internet	61 (31.8)	24 (23.5)	37 (41.1)
Previous pregnancies	17 (8.9)	5 (4.9)	12 (13.3)
Familly, friends	38 (19.8)	12 (11.8)	26 (28.9)
Magazines, brochures	71 (37.0)	33 (32.4)	38 (42.2)
Other	0 (0)	0 (0)	0 (0)
No answer			
No	189 (49.1)	96 (48.5)	93 (49.8)
No answer	4 (1.0)	0 (0)	4 (2.1)
**Did you know that pregnancy makes teeth and gums more vulnerable?**			
Yes	273 (70.9)	124 (62.6)	149 (79.7)
No	104 (27.0)	72 (36.4)	32 (17.1)
No answer	8 (2.1)	2 (1.0)	6 (3.2)

Total number of patients: 385; cohort 1: hospitals of Fribourg and Geneva, number of patients: 198; cohort 2: hospitals of Lausanne and Valais, number of patients: 187.

## Discussion

The aim of the present cross-sectional survey was to explore the oral-health-related knowledge and attitudes of pregnant women attending four general health hospitals located in the French-speaking part of Switzerland. Oral health was considered very important (score 9-10 on the VAS scale) by half of the women (52%) and moderately important by 38% of them. However, only half of them visited their dentist on a routine check-up basis and only 16% consulted a dentist during pregnancy. This proportion could be considered surprisingly low, as similar studies reported a much higher rate of dental consultation during pregnancy. For example, in a recent study by Petit et al^32^ conducted in France, 47% of the women visited their dentist during pregnancy, whereas in other surveys, the attendance varied from 27.3% to 48%.^9,35^ The reasons justifying low attendance were absence of oral problems, large number of medical appointments, and lack of money. More than one-third of the participants (37.5%) did not give a reason for not consulting a dentist during pregnancy. This point, together with the reported relatively low rate of consultation in a routine basis, may be interpreted as lack of interest in oral health.

In our study, 70% of the women did not notice any changes in their oral health condition, which is quite low compared to the existing evidence. The remaining 30% indicated bleeding as the main symptom, followed by gingival redness and oedema. Pain and sensitivity were also reported by 12% and 13% of the women. It is well known that gingival inflammation is a common oral manifestation during pregnancy related to the temporary elevation of sex hormones. As shown in the current literature, approximately 50% of pregnant women report gingival inflammation mainly during the second or third trimester of gestation.^16,26^ A systematic review and meta-analysis concluded that gingival inflammation statistically significantly increased throughout pregnancy and was more pronounced in pregnant women compared to post-partum or non-pregnant women. Inflammation occurred even without plaque accumulation, but probing depth and/or clinical attachment level were not affected.^10,38^

In our study, 89% of the participants reported cleaning their teeth at least twice a day, and almost half of them (45%) also used interdental cleaning aids. These results are slightly higher than those reported in the Swiss Health Survey^36^ on oral hygiene measures and dental visits of the Swiss population: 74% indicated cleaning their teeth or prostheses several times a day, whereas the proportion of respondents using dental floss or toothpicks was also 50%. Moreover, women were found to be more conscientious about oral hygiene than men, as 81.7% women vs 65.5% men reported brushing a few times per day.^36^ The frequency of brushing reported by pregnant participants differs significantly between studies, depending on the country in which the survey is conducted. For example, in a recent study conducted in France, 74% of the participants reported brushing their teeth at least twice per day and only 31% used interdental brushes or floss.^32^ In Spain, brushing twice a day was reported by 79.9% of the participants,^24^ whereas in Saudi Arabia by 33%-51% of those surveyed,^3,11^ and in India by 20.9%.^4^

Only a small proportion of women reported having received a dental check-up during pregnancy. This was mainly due to lack of prevention and information by professionals in charge of pregnancy follow-up. In our study, only 29.1% of participants mentioned that they were informed by their dentist or gynecologist about the importance of oral hygiene during pregnancy, and about half of them (49.9%) were aware that poor oral hygiene can lead to pregnancy complications. These findings are in accordance with the study by Petit et al,^32^ who found that less than 20% of the pregnant women had a discussion about oral health during their gynecological consultations. In another survey including obstetricians/gynecologists in France, although 88% were aware of the inflammatory nature of periodontal diseases and 74.7% were aware of its negative impact on pregnancy outcomes, information on oral health was systematically provided to patients only by 10.5% of them. Furthermore, only 33.2% of practitioners systematically referred a patient to the dentist. Knowledge of periodontal disease and awareness of its negative effects were higher in more experienced practitioners with a private practice and a personal history of periodontal disease.^7^

In the present survey, only 27.6% of respondents mentioned that their doctor or dentist was the source of information on pregnancy complications related to poor oral hygiene; this could be explained by the lack of information and guidelines for dentists as shown in several studies. In a study conducted in Australia, George et al^13^ found that most dentists (99%) stated that pregnant women should receive a dental check-up, yet only 20% agreed that there was good understanding among health professionals on this topic. Most dentists (95.7%) expressed the need for further information about dental care during pregnancy. Another study^19^ addressed to general dentists in Oregon (USA) compared self-reported knowledge and practices with the 2006 guidelines from a New York State Department of Health panel of experts. The study revealed statistically significant differences in the respondents’ attitudes, mainly for items such as obtaining full-mouth radiographs, providing nitrous oxide, administering long-acting anesthetic injections and use of over-the counter pain medications.^19^ A lower knowledge score on oral health care during pregnancy was reported by male dentists than by female dentists.^8^ As for dental hygienists, a survey in North Carolina, USA, showed that the majority (> 80%) correctly identified risk factors such as diabetes and cardiovascular disease, but were less aware in terms of osteoporosis and adverse pregnancy outcomes.^5^ Independent of the years of experience or academic degree, the majority of the dental hygienists (64%) desired more education about dental care for pregnant patients.^34^ A recent survey among Swiss dentists on oral health practices of women during pregnancy also revealed the need for evidence-based guidelines and directions on the best method and time for treatment.^33^

## Conclusion

The findings from this survey suggest that pregnant women in French-speaking Switzerland are moderately informed about the importance of good oral health maintenance during pregnancy to diminish the risks of complications. Furthermore, the active participation of health-care professionals is limited. Health professionals need to more to actively inform pregnant patients about the importance of preventive oral health measures and oral health care during pregnancy. Interdisciplinary education among all health care professionals^2^ remains the most important challenge in order to improve the quality of health care and services.
